# Differential impact of clinicopathological risk factors within the 2 largest ProMisE molecular subgroups of endometrial carcinoma

**DOI:** 10.1371/journal.pone.0253472

**Published:** 2021-09-02

**Authors:** Annukka Pasanen, Mikko Loukovaara, Terhi Ahvenainen, Pia Vahteristo, Ralf Bützow

**Affiliations:** 1 Department of Pathology, University of Helsinki and Helsinki University Hospital, Helsinki, Finland; 2 Applied Tumor Genomics Research Program, University of Helsinki, Helsinki, Finland; 3 Department of Obstetrics and Gynecology, University of Helsinki and Helsinki University Hospital, Helsinki, Finland; 4 Department of Medical and Clinical Genetics, University of Helsinki, Helsinki, Finland; Universita degli Studi di Milano-Bicocca, ITALY

## Abstract

**Objective:**

To assess whether the prognostic impact of conventional risk factors and ancillary biomarkers differs across the 2 largest ProMisE molecular subgroups of endometrial carcinoma (EC).

**Methods:**

Direct sequencing of *POLE* exonuclease domain hot spots and immunohistochemistry for MLH1, PMS2, MSH2, MSH6 and p53 were performed on 745 unselected endometrioid ECs to identify mismatch repair deficient (MMR-D, n = 264) and no specific molecular profile (NSMP, n = 206) ECs. Molecular group-specific survival analyses and interaction analyses were performed to determine the prognostic relevance of clinicopathological factors and various biomarkers (L1 cell adhesion molecule, estrogen and progesterone receptor, beta-catenin, p16, E-cadherin, *KRAS)* within the subgroups.

**Results:**

Molecular subgroup did not have an independent effect on disease-specific survival after adjustment for conventional risk factors (P = 0.101). High grade (G3) and p16 hyperexpression remained significant predictors of survival in NSMP. Stage II-IV, ≥50% myometrial invasion, lymphovascular space invasion and loss of E-cadherin were independent predictors in the MMR-D group. In the interaction analysis, molecular subclass significantly modified the prognostic effect of high grade and p16 hyperexpression, which showed a stronger negative effect on survival in NSMP as compared to MMR-D (P for interaction = 0.016 for grade and 0.033 for p16).

**Conclusions:**

Grade of differentiation and p16 hyperexpression appear to have a stronger prognostic impact in NSMP as compared to MMR-D EC. While these results need to be confirmed in a larger study population, they indicate that differential impact of risk factors needs to be taken into account when developing new molecular class-integrated risk stratification algorithms for EC.

## Introduction

The standard treatment of endometrial carcinoma (EC) consists of hysterectomy and bilateral salpingo-oophorectomy completed with lymph node dissection in selected cases. Post-operative risk stratification guides the selection of adjuvant treatment (observation vs radiotherapy and/or chemotherapy). Current treatment algorithms are based on disease stage, tumor histotype and grade of differentiation, depth of myometrial invasion, lymphovascular space invasion (LVSI) and patient’s age [1].

As histopathology-based risk factors including tumor histotype, degree of differentiation and LVSI, suffer from limited reproducibility [2–4], numerous molecular biomarkers have been tested for EC. In 2013, The Cancer Genome Atlas (TCGA) research network employed extensive molecular information to stratify EC into 4 prognostically distinct subgroups, i.e. polymerase-ϵ (*POLE*) ultramutated, microsatellite instability (MSI) hypermutated, copy-number low, and copy-number high [5]. Due to the high cost and complexity of the methodology used in the TCGA project, more pragmatic diagnostic assays were developed and evaluated in 2 independent EC cohorts, namely, the PORTEC cohort in Leiden [6] and the ProMisE cohort in Vancouver [7]. These molecular classification schemes use surrogate markers for the TCGA subclasses, i.e. targeted *POLE* sequencing for ultramutated subgroup, mismatch repair immunohistochemistry/MSI for hypermutated subgroup and p53 protein expression/mutational analysis for copy-number high subgroup [6, 7]. Tumors lacking any of the above molecular markers are classified as no specific molecular profile (NSMP, surrogate for copy-number low).

Molecular classifiers provide information beyond traditional clinicopathological factors and there is evidence that risk assessment methods can be improved by integrating clinicopathologic and molecular factors [6, 8]. The drastic differences in the frequency of epigenetic events, mutation frequency and stability of the genome suggest distinct pathogenesis of the 4 molecularly defined subgroups. This raises the question whether TCGA-based molecular subgroups should be considered risk factors among others or if they define separate disease entities, within which traditional and novel risk factors should be evaluated. This study investigates the relevance of established clinicopathological factors and ancillary molecular markers within the two largest ProMisE subclasses of EC (MMR-D and NSMP). Interaction effects between molecular group and various risk factors were examined in order to test whether molecular group modifies the effects of individual factors on disease-specific survival.

## Material and methods

This was a retrospective study of 842 patients who underwent primary surgical treatment for stage I-IV EC at the Department of Obstetrics and Gynecology, Helsinki University Hospital between 2007 and 2012. The study was approved by the Institutional Review Board and the National Supervisory Authority for Welfare and Health. Informed consent was waived because of the retrospective nature of the study. All data were fully anonymized before analysis. A tissue microarray of primary tumor samples was constructed during the year 2013 [9]. Follow-up data were last updated in January-March 2018. Histologic slides were reviewed by a gynecological pathologist who marked representative areas of each tumor on 1–2 slides. Four duplicate 0.8-mm cores were drawn from corresponding areas of the paraffin blocks. Given their relative rarity and distinct clinical and prognostic features, non-endometrioid carcinomas were excluded from the study, as were tumors with equivocal morphology. In total, 745 ECs of the endometrioid histotype were included in the study. As only presence/absence of LVSI was reported at the moment of diagnosis and histopathological review, information on the extent of the invasion not available.

Clinicopathologic data were abstracted from institutional medical and pathology records. Stage was determined according to the 2009 International Federation of Gynecology and Obstetrics (FIGO) guidelines [10]. Stage I (disease limited to the uterine corpus) was chosen as the reference level given its superior prognosis compared to EC of stage II and above. The cut-off for age ≥65 years as a risk factor was based on a prior study [11]. Disease-specific survival times were calculated as time from surgery to death from EC. Cause of death was mainly based on medical records. Missing data were complemented from death certificates from Statistics Finland.

The following monoclonal antibodies were used for chromogenic immunohistochemistry on multicore TMA slides: MLH1 (ES05, Dako), MSH2 (G219-1129, BD Biosciences), MSH6 (EPR3945, Abcam), PMS2 (EPR3947, Epitomics), p53 (DO-7, Dako), L1 cell adhesion molecule (L1CAM, SIG-3911, Covance, clone 14.10), beta-catenin (CAT-5H10, Zymed), estrogen receptor alpha (ER, SP1, Roche/Ventana), progesterone receptor (PR, 16, Novocastra), p16 (E6H4, CINtec Histology), E-cadherin (HECD-1, Invitrogen). TMA slides were scanned with 3-dimensional Histech Pannoramic 250 Flash II scanner by Fimmic Oy (Helsinki, Finland). Slide images were managed and analyzed with WebMicroscope Software (Fimmic Oy). A pathologist scored virtual slides blinded to clinical data (A.P). Equivocal cases were examined by a second investigator (R.B) and a consensus was reached.

Mismatch repair protein status was considered deficient when we observed a complete diffuse or clonal loss of nuclear expression in carcinoma cells of 1 or more MMR proteins (MLH1, MSH2, MSH6, PMS2). Abnormal p53 staining was defined as strong and diffuse nuclear staining or completely negative (“null”) staining in carcinoma cells. Weak and heterogeneous staining was classified as wild type expression. Cytoplasmic staining of p53 was not considered as the significance of this finding was unclear at the moment of the scoring. Stromal cells and inflammatory cells served as internal control for MMR and p53 stainings. L1CAM expression was scored as reported earlier, with ≥ 10% of membranous staining considered positive [9]. We adopted a 10% cut-off for ER/PR positivity based on common guidelines for breast cancer and a previous study on EC [12]. As negative p16 was extremely rare (1.7% of the samples), only block type hyperexression of p16 was classified as abnormal staining. For other markers, any nuclear beta-catenin staining and loss of membranous E-cadherin were categorized as abnormal. Samples with scarce carcinoma cells or completely negative staining of the internal control (when applicable) were discarded.

For DNA extraction, representative areas of formalin-fixed paraffin-embedded (FFPE) tumor sections were macrodissected as identified by pathologist assessment. DNA was extracted by proteinase K/phenol-chloroform method. Direct sequencing was performed to identify *POLE* exonuclease domain hot spot mutations in exon 9, exon 13 and exon 14 [13] and *KRAS* mutations in exon 2 (codons 12 and 13). Polymerase chain reaction products were sequenced on an ABI3730xl Automatic DNA Sequencer at the Institute for Molecular Medicine Finland, Helsinki. Sequence graphs were analyzed both manually and with Mutation Surveyor (Softgenetics, State College, PA).

**Fig 1:**
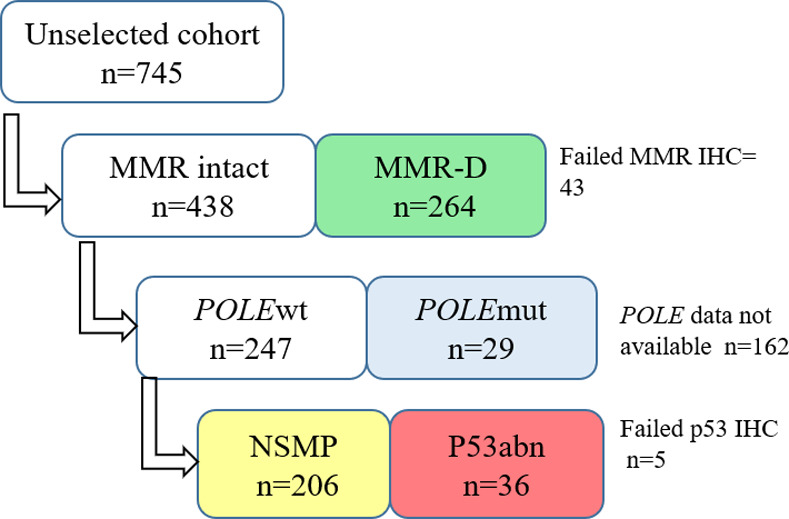
Molecular classification algorithm applied on 535 stage I-IV endometrioid endometrial carcinomas. MMR-D = mismatch repair deficient, wt = wild type, mut = mutated, NSMP = no specific molecular profile, abn = abnormal.

A multistep algorithm analogous to the previously confirmed and externally validated ProMisE classifier [8] was adopted to categorize endometrioid ECs into the following subgroups: MMR-D, *POLE* mutated, p53 abnormal and NSMP (Fig 1). As the sizes of the *POLE* mutated and p53 abnormal groups were insufficient for the a priori defined subgroup analyses, only MMR-D and NSMP cases were included in the study.

Variables selected for statistical analyses were FIGO 2009 stage, age, grade of differentiation, depth of myometrial invasion, LVSI, molecular subgroup (NSMP, MMR-D), L1CAM, ER, PR, beta-catenin, p16, E-cadherin, *KRAS* mutational status and type of adjuvant therapy. Chi-squared test and Fisher exact test (2-sided) were used for comparison of categorical variables. Survival curves were calculated by the Kaplan-Meier method. A log-rank test was used to test for survival differences. Simple and multivariable analyses for prognostic factors were conducted by the Cox proportional hazard model. To test the statistical significance of eventual interaction effects between molecular subgroup and single risk factors, we separately added each interaction term in a multivariable regression model including conventional risk factors and the main effects of the variables forming the interaction term. Statistical significance was set at P < 0.05. Data were analyzed using Statistical Package for Social Sciences (SPSS) version 25 software (IBM Corp., Armonk, NY).

## Results

ProMisE-based molecular classification was successful in 535 patients with stage I-IV endometrioid EC. Of these, 264 (49.3%) were classified as MMR-D, 29 (5.4%) as *POLE* mutated, 36 (6.7%) as p53 abnormal and 206 (38.5%) as NSMP (Fig 1). Clinicopathologic characteristics according to the molecular subgroup are illustrated in Table 1. Patients in the MMR-D group were more likely to be >65 years of age (P = 0.028). Their tumors were more frequently high grade (G3, P<0.001) and displayed more frequent PR negativity (P = 0.011) and E-cadherin loss (P = 0.049) as compared to the NSMP group. Nuclear beta-catenin positivity was more frequent in the NSMP group (P<0.001).

**Table 1:** Clinicopathologic characteristics and molecular features according to the molecular subgroup in stage I-IV endometrioid endometrial carcinoma (n = 470).

	NSMP (n = 206)	MMR-D (n = 264)	P
	n, (%)	n, (%)	
Age >65 years	108/206 (52.4)	165/264 (62.5)	**0.028**
ESMO-ESGO-ESTRO risk groups			0.134
Low risk	110/206 (53.4)	110/264 (41.7)	
Intermediate risk	24/206 (11.7)	32/264 (12.1)	
High-intermediate risk	23/206 (11.2)	39/264 (14.8)	
High risk stage I-II	23/206 (11.2)	37/264 (14.0)	
High risk stage III-IV	26/206 (12.6)	46/264 (17.4)	
Grade 3	13/206 (6.3)	54/264 (20.5)	**<0.001**
Myometrial invasion ≥50%	76/206 (36.9)	108/264 (40.9)	0.376
Lymphovascular invasion +	43/206 (20.9)	68/264 (25.8)	0.216
L1CAM positivity (≥10%)	8/198 (4.0)	17/254 (6.7)	0.221
ER negativity (<10%)	13/201 (6.5)	25/250 (10.0)	0.179
PR negativity (<10%)	25/204 (12.3)	53/249 (21.3)	**0.011**
Nuclear beta-catenin	44/206 (21.4)	18/260 (6.9)	**<0.001**
p16 hyperexpression	9/206 (4.4)	21/258 (8.1)	0.101
E-cadherin loss	2/204 (1.0)	10/254 (3.9)	**0.049**
k-ras mutation	32/177 (18.1)	44/195 (22.6)	0.284
Adjuvant therapy			0.486
No adjuvant therapy	30/206 (14.6)	35/264 (13.3)	
Vaginal brachytherapy	116/206 (56.3)	128/264 (48.5)	
WPR	28/206 (13.6)	46//264 (17.4)	
Chemotherapy	11/206 (5.3)	17/264 (6.4)	
Chemotherapy and WPR	21/206 (10.2)	38/264 (14.4)	

*POLE* mutational data was not available for 162 patients with MMR intact tumors. These cases were excluded from further analysis. When comparing the successfully classified NSMP cases and MMR intact–p53 wild type cases that were excluded due to missing *POLE* data (mainly consisting of excluded NSMP cases, given the low prevalence of *POLE* mutation), the distribution of clinicopathological features did not significantly differ between the included and excluded cases with the exception of myometrial invasion (S1 Table).

**Fig 2:**
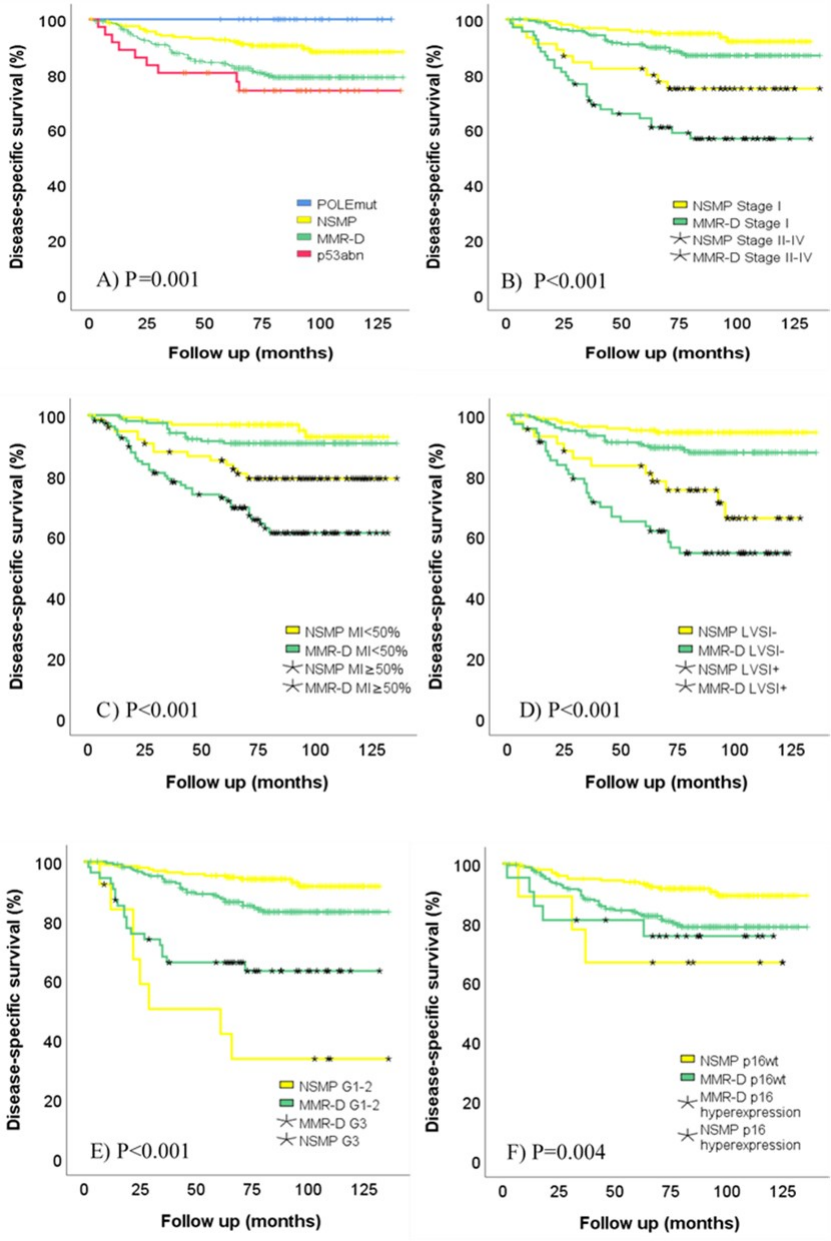
Kaplan-Meier curves for disease-specific survival according to TCGA-based molecular subgroups and single risk factors within NSMP/MMR-D subgroups: A) Molecular classification, B) FIGO 2009 stage, C) Myometrial invasion (MI), D) Lymphovascular space invasion (LVSI), E) Grade of differentiation, F) p16 expression. POLEmut = POLE mutated, NSMP = no specific molecular profile (yellow), MMR-D = mismatch repair deficient (green), p53abn = p53 abnormal.

Median follow-up time was 83 months (range 1–136 months). Seventy-two patients (15.3%) died of EC during follow-up. Disease-specific mortality rate was 10.2% in the NSMP and 19.3% in the MMR-D group (P = 0.001). Kaplan-Meier disease-specific survival curves according to molecular subgroups are depicted in Fig 2A. Survival curves mirrored the original TCGA-data with the *POLE*mut group having the best prognosis followed by NSMP, MMR-D and p53abn (P = 0.001). In pairwise comparisons between NSMP and MMR-D, survival difference remained significant (P = 0.005). Survival curves displaying the effect of single risk factors within the molecular subgroups are depicted in Fig 2B–2F. High grade and p16 hyperexpression appear to have a particularly strong negative prognostic effect on NSMP as compared to MMR-D cases (Fig 2E and 2F, respectively).

The results of simple and adjusted Cox regression survival analyses in NSMP and MMR-D groups are shown in Table 2. Risk factors presenting statistical significance (P<0.05) in univariate analysis were included in multivariable regression analysis performed separately for each molecular subclass. In the NSMP group, high grade (HR = 7.2, P = 0.047) and p16 (HR = 6.7, P = 0.024) remained significant independent predictors (Table 2). In the MMR-D group, the effect of advanced stage (HR = 2.3, P = 0.047), deep myometrial invasion (HR = 2.1, P = 0.036), LVSI (HR = 2.8, P = 0.002), and E-cadherin loss (HR = 5.1, P = 0.012) remained significant in multivariable analysis (Table 2).

**Table 2:** Subgroup-specific simple and multivariable Cox regression analysis of disease-specific survival in NSMP (n = 206) and MMR-D (n = 264) stage I-IV EC.

	Simple HR (95% CI)	P	Adjusted HR (95% CI)	P
**Stage II-IV** (vs stage I)				
NSMP	7.6 (3.2–17.8)	**<0.001**	1.4 (0.2–19.9)	0.747
MMR-D	4.1 (2.3–7.1)	**<0.001**	2.3 (1.0–5.3)	**0.047**
**Grade 3** (vs grade 1–2)				
NSMP	14.2 (5.9–34.7)	**<0.001**	7.2 (1.0–51.0)	**0.047**
MMR-D	3.0 (1.7–5.3)	**<0.001**	1.6 (0.8–3.1)	0.191
**Myometrial invasion ≥50%** (vs <50%)				
NSMP	4.6 (1.8–12.0)	**0.002**	3.4 (0.8–14.6)	0.094
MMR-D	4.7 (2.5–8.6)	**<0.001**	2.1 (1.0–4.2)	**0.036**
**LVSI+** (vs no LVSI)				
NSMP	5.7 (2.4–13.5)	**<0.001**	2.4 (0.7–8.0)	0.124
MMR-D	4.6 (2.6–7.9)	**<0.001**	2.8 (1.5–5.4)	**0.002**
**L1CAM ≥10%** (vs <10%)				
NSMP	9.8 (3.2–30.0)	**<0.001**	(0.140–9.11)	0.910
MMR-D	2.6 (1.1–6.0)	**0.031**	2.2 (0.8–6.1)	0.128
**ER <10%** (vs ≥10%)				
NSMP	8.3 (3.2–21.6)	**<0.001**	0.5 (0.1–4.6)	0.551
MMR-D	2.2 (1.0–4.7)	**0.043**	1.0 (0.4–2.6)	0.951
**PR <10%** (vs ≥10%)				
NSMP	2.8 (1.0–7.9)	**0.045**	1.8 (0.4–9.3)	0.463
MMR-D	1.2 (0.6–2.3)	0.667	NA	NA
**p16 hyperexpression** (vs focal or negative)				
NSMP	4.2 (1.2–14.2)	**0.022**	6.7 (1.3–34.7)	**0.024**
MMR-D	1.3 (0.5–3.3)	0.550	NA	NA
**E-cadherin loss** (vs intact)*				
MMR-D	3.0 (1.1–8.4)	**0.034**	5.1 (1.4–18.3)	**0.012**
**Nuclear beta-catenin** (vs membranous)				
NSMP	0.4 (0.9–1.6)	0.182	NA	NA
MMR-D	1.6 (0.6–4.1)	0.347	NA	NA
***KRAS* mutation** (vs wild type)				
NSMP	0.8 (0.2–2.9)	0.786	NA	NA
MMR-D	1.1 (0.5–2.3)	0.767	NA	NA
**Adjuvant therapy** (vs none/brachytherapy)				
NSMP				
WPR	2.0 (0.5–7.5)	0.313	0.5 (0.1–4.3)	0.533
Chemotherapy	6.4 (1.7–23.6)	**0.007**	2.4 (0.3–21.1)	0.441
Chemotherapy and WPR	6.7 (2.4–18.5)	**<0.001**	1.0 (0.1–10.7)	0.996
MMR-D				
WPR	3.9 (1.9–8.0)	**<0.001**	1.4 (0.5–3.6)	0.498
Chemotherapy	4.7 (1.8–12.1)	**0.001**	1.5 (0.4–5.3)	0.544
Chemotherapy and WPR	5.6 (2.7–11.2)	**<0.001**	1.5 (0.5–4.0)	0.464

*Not analyzed in NSMP (due to low prevalence, n = 2). NSMP = no specific molecular profile, MMR-D = mismatch repair deficient, L1CAM = L1 cell adhesion molecule, ER = estrogen receptor, PR = progesterone receptor, WPR = whole pelvic radiotherapy, NA = not analyzed. Bolded *P*-values indicate statistical significance (P<0.05).

In the interaction analysis, the prognostic effect of grade of differentiation and p16 expression differed significantly between the molecular groups (HR = 0.3, P = 0.016 for the interaction term molecular group*grade and HR = 0.2, P = 0.033 for the interaction term molecular group*p16, Table 3). This result indicates that the prognostic impact of these 2 factors was significantly modified by the molecular group. As regards the effect of disease stage, myometrial invasion and LVSI, we were not able to demonstrate statistically significant differences between the molecular groups. After adjusting for clinicopathological risk factors, the effect of molecular group became non-significant (P = 0.101, Table 3).

**Table 3:** Multivariable Cox regression analysis of disease-specific survival in stage I-IV NSMP/MMR-D endometrial carcinoma (n = 470).

	HR (95% CI)	P
Stage I	1	
Stage II-IV	1.6 (1.0–2.6)	0.217
Grade 1–2	1	
Grade 3	2.4 (1.4–4.1)	**0.002**
Myometrial invasion <50%	1	
Myometrial invasion ≥50%	2.3 (1.3–4.1)	**0.006**
No lymphovascular invasion	1	
Lymphovascular invasion +	12.7 (1.6–4.6)	**<0.001**
NSMP	1	0.101
MMR-D	1.6 (1.0–2.6)	
Adjuvant therapy		
No therapy or brachytherapy	1	
WPR	1.1 (0.5–2.4)	0.719
Chemotherapy	2.2 (0.9–5.7)	0.095
Chemotherapy and WPR	1.6 (0.7–3.7)	0.316
Interaction terms		
Molecular group*grade	0.3 (0.1–0.8)	**0.016**
Molecular group*p16	0.2 (0.0–0.9)	**0.033**

NSMP = no specific molecular profile, MMR-D = mismatch repair deficient, WPR = whole pelvic radiotherapy. Bolded.

*P*-values indicate statistical significance (P<0.05).

## Discussion

The advent of the TCGA molecular characterization of EC [5] has raised an interest to integrate traditional risk factors and molecular classification methods in order to provide more objective and reproducible risk-assessment [14]. Molecular classifiers provide independent prognostic information in EC, but they also correlate with traditional clinicopathological factors. For example, within the molecular subclasses, p53 abnormal and MMR-D ECs more frequently present aggressive features such as deep myometrial invasion, LVSI and high-grade histology [8]. However, this correlation is incomplete: e.g. p53 abnormal tumors are frequently of serous subtype, but may also be endometrioid, NSMP tumors are often low-grade endometrioid ECs, but may also be high-grade tumors. In addition to divergent clinicopathological profiles, molecular subgroups have many other distinctive features. For example, the prognostic effect of old age, overweight/obesity and type 2 diabetes are not uniform across molecular subgroups [15]. The frequency of homologous recombination deficiency and the expression of immunotherapy target molecules programmed death 1 (PD-1) and its ligand 1 (PD-L1) vary according to molecular subgroups [13, 16, 17]. Further, the response to adjuvant radiotherapy appears dissimilar for the subgroups [18, 19]. These findings suggest that integrating molecular information into the EC risk stratification algorithms may improve the adjuvant treatment selection.

In a meta-analysis including pooled data from 6 TCGA classification-based studies, MMR-D showed a risk of death from EC about twice as high as NSMP in unadjusted analysis [20]. Survival differences became non-significant after adjustment for clinicopathological factors both in the pooled analysis and in the present study [20]. Thus, the prognostic effect of MMR-D appears to be related to the presence of conventional risk factors [8]. In order to formulate optimal treatment algorithms, we need to determine whether the impact of each risk factor varies across the molecular subgroups and if so, the relative weight that each factor should assume in the stratification algorithm. In fact, this and previous studies provide preliminary evidence that the effect of risk factors may not be uniform across the molecular subclasses of EC. In our study, interaction testing suggested a modifying effect of the molecular group as regards the prognostic impact of grade of differentiation and p16 expression, which both had a stronger effect on survival in NSMP than in MMR-D cases. Accordingly, in a previous study conducted on G3 endometrioid EC, NSMP showed worse survival compared to MMR-D, while the opposite was true when endometrioid ECs of all grades of differentiation were included in the survival analysis [6, 21]. By contrast, *POLE*mut EC typically presents an indolent clinical course regardless of the relatively frequent G3 histotype [5]. Interestingly, a recent study suggested a correlation between strong p16 hyperexpression and poor survival in endometrioid and clear cell ovarian carcinoma [22]. Further, in our study, E-cadherin loss was extremely rare in NSMP, but it independently predicted outcome in MMR-D. Complementary studies are needed to clarify the role of p16 and E-cadherin in EC, even though their low prevalence in endometrioid EC may limit their use in clinical practice.

The ongoing PORTEC-4a trial combines TCGA-based molecular subgroups, clinicopathological risk factors and ancillary molecular markers in order to investigate standard versus molecular-based recommendation for radiotherapy in early-stage EC. The trial algorithm subcategorizes ECs according to the presence of high-risk features (abnormal p53, LVSI, L1CAM positivity), intermediate risk (MSI and *CTNNB1* mutation in the NSMP group) and low-risk features (*POLE* mutation and absence of *CTNNB1* mutation in NSMP) [23]. L1CAM is a promising biomarker that large retrospective studies have found to predict poor outcome in EC [9, 24–26]. In the present study, L1CAM positivity was associated with poor outcome in both NSMP and MMR-D EC and molecular subclass did not significantly modify the prognostic effect. *CTNNB1* mutational data was not available and immunohistochemical positivity for beta-catenin appeared not to have prognostic value. Regarding other potential biomarkers included in our study *KRAS* mutation did not correlate with outcome and hormone receptor status lost its significance after adjusting for confounding variables.

A strength of this study is our regularly updated database with long follow-up time. Comprehensive reports on causes of death allowed us to investigate disease-specific survival instead of overall survival, which is a less accurate measure of outcome when searching for causalities in cancer patients. A limitation was the relatively high rate of missing *POLE* data, which was due to DNA not being available, limited yields of high-quality DNA from formalin-fixed tissue and our stringent criteria of inclusion for *POLE* sequencing data. Given the order of decision steps in our molecular classification algorithm, missing *POLE* data limited the number of patients in NSMP but not in the MMR-D subgroup, resulting in a higher than expected proportion of MMR-D cases. However, the distribution of clinicopathological characteristics in the NSMP group remained representative. Given the profound pathogenetic and prognostic differences between endometrioid and non-endometrioid carcinomas of the uterus, analyses were restricted to the more common endometrioid subtype of endometrial carcinoma.

Intratumoral heterogeneity of protein expression may lead to decreased sensitivity in TMA studies. Clonal loss of MMR protein expression has been reported but this phenomenon is not common. As the rate of mismatch repair deficiency in our study was not lower than generally reported, our results were unlikely to be compromised by false negative scores [8, 27]. To overcome the problem of false positivity such as apparent MMR protein loss due to poor fixation, we strictly reported MMR deficiency only for cases showing adequate staining of the internal control. In addition, previous studies have shown that TMAs with three core biopsies per tumor adequately represent the tumor phenotype, even with antigens known to be heterogeneous [28, 29]. To improve sensitivity, we included 4 tissue cores from each tumor in our TMA. We have previously demonstrated a high concordance between our TMA and the corresponding whole sections, as concerns expression of L1CAM, a highly heterogeneous antigen [9]. As an advantage, TMA methodology allowed us to analyze a large number of cases.

This proof-of-concept study provides interaction analysis-based data which show that the relative weights of different risk factors vary between molecular subgroups (NSMP, MMR-D) of EC. Accordingly, novel putative disease markers of EC should be analyzed in a molecular subgroup-specific manner. Larger studies are needed to confirm our findings and to reveal further interaction effects for known risk factors that we may have missed due to limited sample size. Eventually, randomized clinical trials incorporating both molecular and clinicopathological features in treatment algorithms will determine the true value of TCGA-integrated classifiers and optimal algorithms to guide the management of EC patients. S1 Table. Distribution of clinicopathological characteristics in NSMP and excludedf MMRwt/p53wt/POLE unknown cases

## Supporting information

S1 Table Distribution of clinicopathological characteristics in NSMP and excluded MMRwt/p53wt/*POLE* unknown cases. (DOCX)
