# Supplementary material for: Differential impact of clinicopathological risk factors within the 2 largest ProMisE molecular subgroups of endometrial carcinoma
Source: PLoS One. 2021 Sep 2;16(9):e0253472. doi: 10.1371/journal.pone.0253472 (PMC8412344; doi:10.1371/journal.pone.0253472)
Supplement: S1 Table — (DOCX) [file pone.0253472.s001.docx]

S1 Table. Distribution of clinicopathological characteristics

in NSMP and excluded MMRwt/p53wt/*POLE* unknown cases

|  | NSMP  (n=206) | Missing *POLE*  (n=141) | P |
| --- | --- | --- | --- |
| Age >65 years | 108/206 (52.4) | 66/141 (46.8) | 0.304 |
| ESMO-ESGO-ESTRO  Low risk  Intermediate risk  High-intermediate risk  High risk stage I-II  High risk stage III-IV | 110/206 (53.4)  24/206 (11.7)  23/206 (11.2)  23/206 (11.2)  26/206 (12.6) | 86/136 (63.2)  17/136 (12.5)  18/136 (13.2)  7/136 (5.1)  8/136 (5.9) | 0.065 |
| Grade 3 | 13/206 (6.3) | 11/141 (7.8) | 0.591 |
| Myometrial invasion ≥50% | 76/206 (36.9) | 34/141 (24.1) | **0.012** |
| Lymphovascular invasion + | 43/206 (20.9) | 18/134 (13.4) | 0.081 |

NSMP=no specific molecular profile, MMR=mismatch repair, wt=wild type
